# Case Report: A case of delayed-onset benign distal esophageal stricture after Yttrium-90 microsphere selective internal radiation therapy

**DOI:** 10.3389/fonc.2026.1928806

**Published:** 2026-09-29

**Authors:** Yang Gao, Yao Wei, Hengxiao Lu, Linlin Ji

**Affiliations:** 1 Department of Thoracic Surgery, Weifang People's Hospital, The First Affiliated Hospital of Shandong Second Medical University, Weifang, China; 2 Department of Radiation Oncology, Weifang Traditional Chinese Hospital, Weifang, China

**Keywords:** benign esophageal stricture, dysphagia, hepatocellular carcinoma, radiation-induced injury, Y90-SIRT

## Abstract

Yttrium-90 microsphere selective internal radiation therapy (Y90-SIRT) is a common local interventional treatment for hepatocellular Carcinoma and liver metastases. With the increasing use, extrahepatic complications have been gradually recognized. We report a case of a 47-year-old patient with hepatocellular carcinoma (HCC) who gradually developed distal esophageal ulcers and subsequent benign cicatricial stricture, clinically presumed to be radiation-induced, following Y90-SIRT treatment. Concurrent lenvatinib may impair mucosal repair function. This rare sequela emphasizes rigorous preoperative vascular evaluation, long-term monitoring and timely intervention, particularly for patients undergoing combined targeted treatment.

## Introduction

Yttrium-90 selective internal radiation therapy (Y90-SIRT) is a widely used locoregional treatment for hepatocellular carcinoma and liver metastases (1, 2). Along with its expanding clinical application, extrahepatic adverse events have gained increasing attention. Gastrointestinal radiation injury is a well-documented complication of Y90-SIRT, predominantly manifesting as acute mucosal ulcers (3, 4), whereas delayed cicatricial stricture of the distal esophagus remains extremely rare and easily misdiagnosed clinically. At present, the clinical characteristics, high-risk factors and optimal preventive strategies for this rare long-term sequela have not been fully clarified. Moreover, whether concurrent targeted therapy may exacerbate esophageal mucosal injury and participate in disease progression still lacks sufficient clinical evidence (5, 6). Herein, we report a hepatocellular carcinoma patient with delayed distal esophageal stricture after Y90-SIRT. We summarize clinical and pathological features and discuss relevant clinical strategies.

## Case presentation

A 47-year-old male presented to the hospital with a 3-day history of abdominal pain. Contrast-enhanced abdominal CT showed multiple solid space-occupying liver lesions, complicated with cirrhosis, splenomegaly, portal hypertension, and lower esophageal varices. Gastroscopy revealed distal esophageal and gastric mucosal congestion and edema (**Figure 1A**). Liver lesion biopsy confirmed well-differentiated hepatocellular carcinoma (HCC), staged cT2N0M0 (CNLC IIB). Y90-SIRT was first administered due to multiple intrahepatic nodules, followed by surgical resection evaluation.

**Figure 1:**
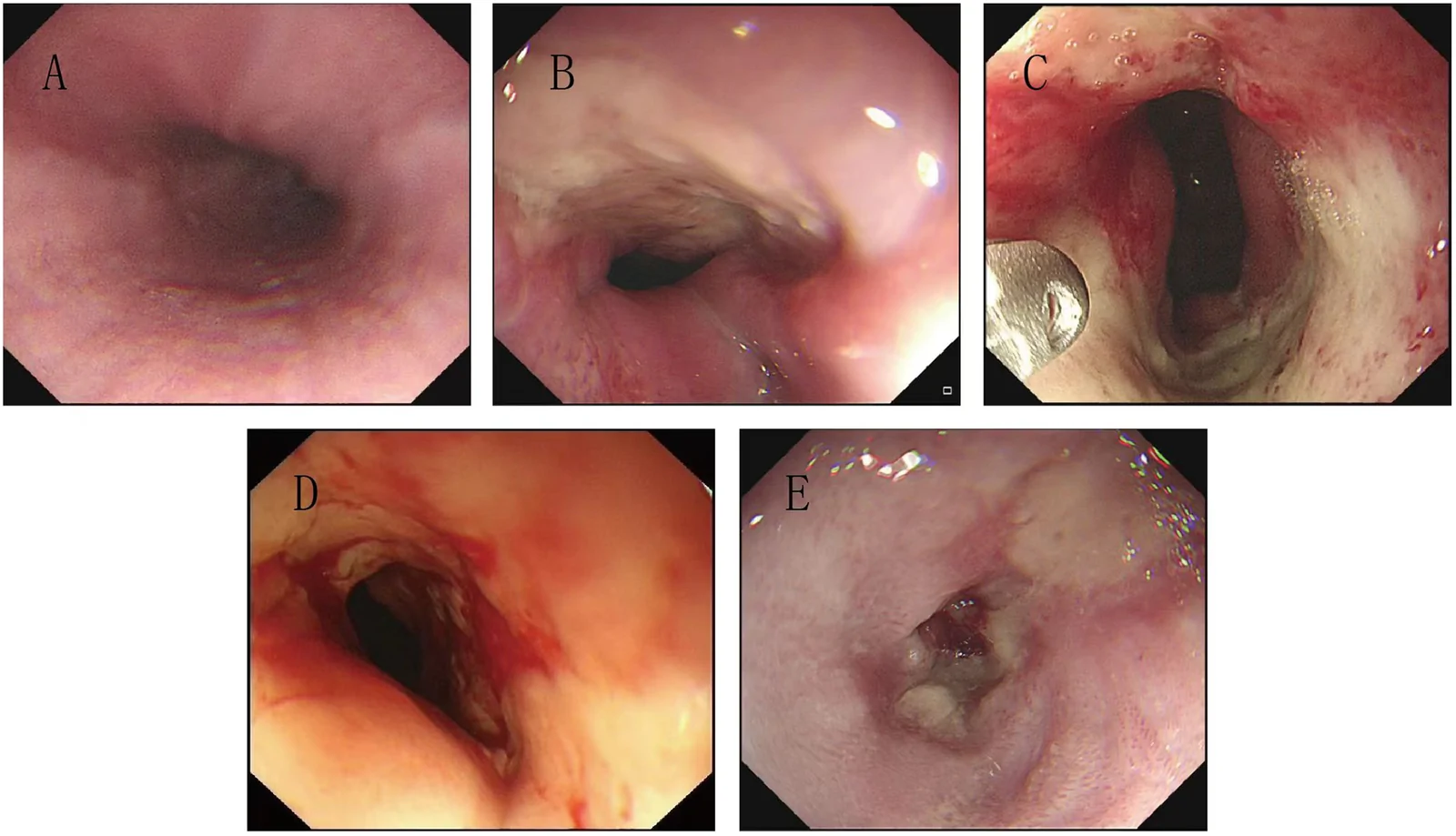
**(A)** Gastroscopy prior to Y90-SIRT. **(B)** Gastroscopy at 3 months after Y90-SIRT. **(C)** Gastroscopy at 7 months after Y90-SIRT. **(D)** Gastroscopy at 12 months after Y90-SIRT. **(E)** Gastroscopy at 16 months after Y90-SIRT.

Pre-Y90 hepatic arteriography was performed via right femoral artery puncture. The initial celiac trunk and superior mesenteric arteriography was performed to screen for anomalous non-anatomical extrahepatic arterial supply, and no such aberrant feeders were identified. Technetium-99 injection and postoperative ECT/CT showed intrahepatic tumor radioactive concentration without extrahepatic uptake (**Figures 2A, B**). Subsequently, selective angiography of the hepatic arterial branches, combined with cone-beam CT (CBCT) three-dimensional reconstruction, was performed to precisely delineate the tumor-feeding vessels. Y90 resin microsphere endovascular brachytherapy combined with transcatheter embolization was then carried out. This detailed evaluation identified extensive tumor staining in the left hepatic lobe, supplied by the left hepatic artery, left gastric artery branches, and left inferior phrenic artery; multiple punctate tumor staining was seen in the right hepatic lobe, supplied by the right hepatic artery. The left inferior phrenic artery was embolized with gelatin sponge particles to devascularize its tumor-feeding branches. Under CBCT guidance, microcatheters were selectively inserted into tumor-feeding arteries. Based on the tumor volume and vascular territory supplied by each feeding artery, the activity was allocated as follows: the left gastric artery branches supplied approximately 85% of the total target tumor volume, receiving 3.0 GBq, while the left hepatic artery branches received 0.5 GBq. During LGA catheterization, the microcatheter was positioned distally within the tumor-feeding sub-branches; no visible esophageal branches were identified from the targeted segment, and slow pulsed manual injections were performed under fluoroscopic monitoring to minimize reflux. Subsequently, lipiodol embolization was performed on the right hepatic artery. Postoperatively, ECT/CT imaging revealed predominant radioactive concentration within the hepatic tumors. Upon detailed review of the fused Bremsstrahlung SPECT/CT images, a faint Y 90 radioactivity was observed immediately adjacent to the anterior and left wall of the distal esophagus (**Figure 2D**), which was Inherently indistinguishable from the intrahepatic Y 90 radioactivity on 2D planar images (**Figure 2C**). The patient was discharged with oral lenvatinib and tenofovir. After discharge, the patient received oral lenvatinib at 8 mg daily as postoperative adjuvant therapy and remained on this dose without interruption throughout the follow-up period. Three months after the Y90-SIRT treatment, laparoscopic hepatectomy plus radiofrequency ablation was performed. Postoperative pathology showed two liver masses: one with 65% massive necrosis and 25% residual well-differentiated HCC, and 10% tumor stroma, negative margin; the other with massive hepatocyte necrosis without residual carcinoma, indicating a favorable tumor response despite the subsequent esophageal complication.

**Figure 2:**
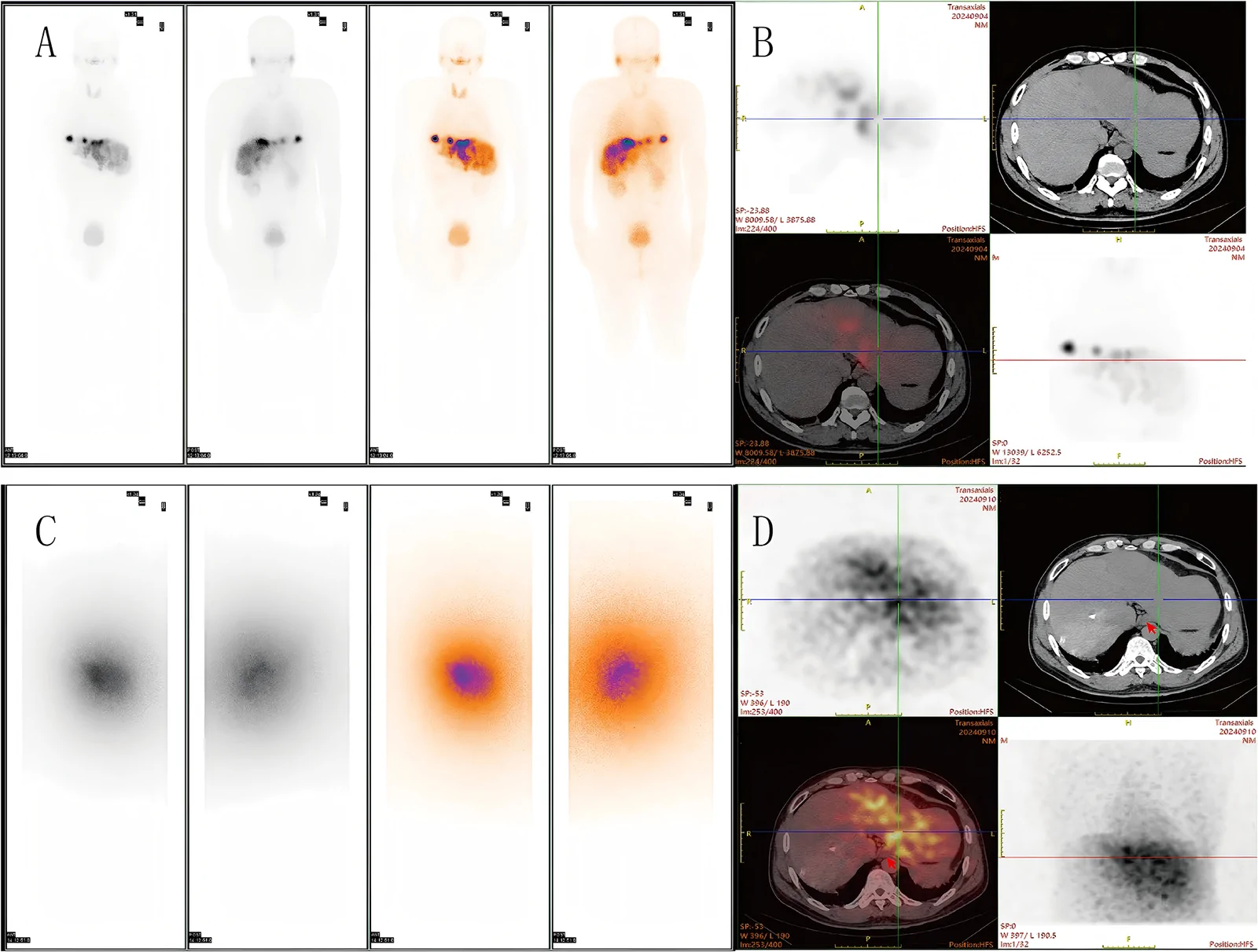
Pre-treatment ^99m^Tc-MAA SPECT/CT and post-treatment bremsstrahlung SPECT/CT. **(A)** Pre-treatment whole-body scan showing radiotracer concentration within the hepatic tumors. **(B)** Pre-treatment axial image at the level of the liver tumors. **(C)** Post-treatment whole-body scan. The intense hepatic activity partially obscures soft tissue contours, an inherent limitation of bremsstrahlung imaging. **(D)** Post-treatment axial fusion image. A red arrow indicates the region adjacent to the distal esophageal wall. A faint and ill-defined signal is observable in this area, which largely overlaps with the adjacent hepatic activity and scatter background, making it indistinguishable from intrahepatic signal on planar imaging.

The patient developed mild dysphagia with odynophagia at 3 months after Y90 treatment, which gradually worsened over time. Serial follow-up endoscopies were performed to monitor the progressive esophageal lesions. Four follow-up gastroscopies at different stages showed progressive distal esophageal lesions: 3 month after Y90 treatment, the first revealed a large infiltrative ulcer (1/2 circumference), pathology showing distal esophageal ulcer (**Figure 1B**); 7 months after Y90 treatment, the second showed a circumferential ulcer extending to the cardia, pathology revealing distal esophageal ulcer with surrounding squamous epithelial papillomatous hyperplasia and acute-chronic inflammation (**Figure 1C**); 12 months after Y90 treatment, the third showed significant stenosis with ulcer, pathology showing distal esophageal ulcer with inflammatory exudation, epithelial hyperplasia, and focal proliferative myofibroblasts (**Figure 1D**); 16 months after Y90 treatment, the fourth showed severe ulcerative stenosis (endoscope impassable), which was clinically presumed to be benign esophageal stricture secondary to probable radiation-induced injury (**Figure 1E**). Subsequent interventional therapy: upper gastrointestinal radiography and esophageal balloon dilatation were performed under local anesthesia, which significantly relieved stenosis and improved the patient’s dysphagia. Although endoscopic biopsies did not identify microspheres in the wall of esophagus, the combined observations on faint Y-90 immediately adjacent to the wall of esophagus, the temporal relationship, and progressive endoscopic changes, all were consistent with radiation-induced injury as one of primary etiology.

## Discussion

Gastrointestinal complications associated with Y90-SIRT are predominantly acute radiation-induced ulcers, while delayed benign stricture of the distal esophagus is extremely rare and is easily misdiagnosed clinically as local tumor invasion or metastasis. Unlike ordinary mucosal ulcers, radiation-induced gastrointestinal ulcerative injuries tend to form extensive fibrous scar tissue through repeated cycles of injury and repair, ultimately resulting in luminal contracture and stricture.

Ectopic microsphere reflux into the distal esophageal feeding arteries represents the most plausible cause of radiation-induced esophageal injury and subsequent cicatricial stricture in this case. Anatomically, the left gastric artery gives off esophageal branches that directly perfuse the distal esophagus and gastroesophageal junction, representing the conventional and non-anomalous blood supply to this region. Therefore, when Y90 microspheres are infused via the left gastric artery branches—as in the present case—there exists a plausible anatomical conduit for microscopic ectopic reflux into the distal esophageal vasculature, potentially leading to radiation-induced mucosal injury and subsequent cicatricial stricture. Of note, although the left gastric artery and left inferior phrenic artery are anatomically extrahepatic branches, they were prospectively identified as tumor feeders during the pre-procedural CBCT-based three-dimensional vascular mapping, rather than being unanticipated intraoperative discoveries. Nevertheless, the occurrence of delayed esophageal stricture in this patient suggests that even with pre-procedural identification, microscopic microsphere reflux via the left gastric artery may not be entirely preventable, underscoring the value of rigorous preoperative multimodal vascular evaluation and long-term post-treatment surveillance.

The patient’s tumor-feeding arteries included the left hepatic artery, left gastric artery, and left inferior phrenic artery. Intraoperatively, prophylactic embolization of the left inferior phrenic artery was performed without Y90 infusion. Y90 microspheres were delivered at 3 GBq via tumor branches of the left gastric artery and 0.5 GBq via branches of the left hepatic artery. Anatomically, the left gastric artery naturally supplies the distal esophagus. It is thus hypothesized that the delayed distal esophageal ulcer and stricture may be attributable to microscopic ectopic reflux of Y90 microspheres from the left gastric artery with subsequent mucosal involvement.

Admittedly, endoscopic biopsies did not identify microspheres, and the post procedural SPECT/CT showed a faint, periesophageal Y-90 radioactivity, direct confirmatory evidence of ectopic microsphere deposition remains insufficient. The limited sensitivity and uncertainties in Y-90 dose estimation based on bremsstrahlung SPECT/CT are well recognized in the literature: one study reported a sensitivity of only 13% for predicting gastrointestinal ulceration following Y90 radioembolization (7).recognizeliter

Despite the lack of direct imaging or histopathological confirmation, several lines of indirect evidence collectively support this hypothesis. The temporal progression from an infiltrative ulcer at 3 months to circumferential ulceration at 7 months and eventually to severe cicatricial stenosis at 16 months is consistent with a chronic radiation-induced fibrotic process. The pathological evolution—from inflammatory exudation to prominent myofibroblastic proliferation—mirrors the typical course of radiation-induced tissue repair and scarring. And the anatomical connection between the left gastric artery and the distal esophageal vascular bed provides a plausible conduit for microsphere migration. Taken together, while definitive proof of ectopic microsphere deposition is lacking, the collective clinical, anatomical, and temporal evidence strongly suggests this mechanism as the most probable explanation for the patient’s delayed esophageal stricture.

The patient received long-term oral lenvatinib for postoperative antitumor therapy. As demonstrated in pharmacological and preclinical studies (8, 9), lenvatinib is a multi-target tyrosine kinase inhibitor that suppresses VEGFR and FGFR signaling, with documented anti-angiogenic effects on vascular endothelium. This mechanism may theoretically reduce mucosal perfusion and impair the self-repair capacity of the esophageal mucosa, thereby carrying a potential risk of inducing or exacerbating drug-related esophagitis. With a similar duration of lenvatinib administration in our case, the esophageal lesion progressed progressively from ulceration to substantial fibrous hyperplasia and severe luminal stricture—a pattern that is far more severe and qualitatively different from lenvatinib-associated esophageal injuries reported previously, including cases of lenvatinib monotherapy that resulted in acute esophageal fistula formation (10), as well as the esophagitis described in a patient receiving lenvatinib plus pembrolizumab (11). While the latter case is confounded by the concurrent use of immunotherapy and thus cannot be attributed solely to lenvatinib, both reported injuries are predominantly acute and inflammatory in nature, contrasting with the delayed-onset, progressively fibrotic and cicatricial pattern observed in our patient.

In addition to the above mechanistic considerations regarding lenvatinib, it is also critical to acknowledge that other concurrent clinical confounders may have independently or synergistically contributed to the progression from acute ulcer to recalcitrant cicatricial stricture. First, the patient had significant baseline portal hypertension with distal esophageal varices and mucosal congestion, indicating pre-existing chronic microvascular ischemia and compromised epithelial barrier function in the distal esophagus, which likely lowered the local tolerance to any additional insult. Second, laparoscopic hepatectomy performed 3 months post-Y90 may have introduced additional perioperative stressors—including hemodynamic fluctuations, anesthesia-related gastroesophageal reflux, or postoperative systemic inflammation—that could temporarily impair mucosal perfusion and delay ulcer healing during a critical window of tissue repair. Thus, rather than acting as a sole driver, radiation injury probably served as the initial “trigger,” while the interplay of lenvatinib-mediated anti-angiogenic effects, portal hypertensive vasculopathy, and surgical stress likely created a permissive microenvironment that perpetuated mucosal breakdown and promoted excessive fibrogenesis. This multifactorial perspective does not weaken the radiation hypothesis but rather refines it, acknowledging that the clinical phenotype observed reflects a convergence of multiple pathogenic hits in a vulnerable host.

## Conclusion

Delayed benign stricture of the distal esophagus represents a rare but severe long-term complication following Y90-SIRT, which may still develop even in patients with unremarkable preoperative MAA scans. Based on our clinical reasoning, persistent radiation injury secondary to presumed ectopic Y90 microsphere deposition remains the most probable primary trigger. However, the concurrent contributions of continuous lenvatinib therapy, portal hypertensive vasculopathy, and hepatectomy-related perioperative stress should not be overlooked, as they may have synergistically exacerbated mucosal breakdown and fibrotic progression. While definitive confirmatory evidence of microsphere deposition was not obtained in this case, the collective clinical, anatomical, and temporal evidence supports radiation injury as the dominant etiology, with lenvatinib playing a potential adjunctive role in impairing mucosal repair rather than serving as an independent cause of the stricture. Long-term endoscopic surveillance is recommended for Y90-SIRT recipients even in the absence of acute postoperative gastrointestinal symptoms. This case underscores the critical importance of a rigorous preoperative vascular evaluation. The selective angiography of target vessels should be meticulously scrutinized for any non-target branches, such as the accessory left gastric artery (12). When such anatomy is identified or suspected, cone-beam CT (CBCT) is a valuable adjunct to confirm the vascular anatomy. Subsequently, superselective prophylactic embolization of these high-risk branches using microcatheters and coils may be considered to prevent microsphere reflux, although some studies suggest that in experienced hands, omitting coiling could be a safe alternative (13). Post-embolization angiography and long-term endoscopic surveillance are recommended to confirm efficacy and monitor for delayed complications.

## Data Availability

The original contributions presented in the study are included in the article/supplementary material. Further inquiries can be directed to the corresponding author.
